# Ring Chromosome 13 and Ambiguous Genitalia

**DOI:** 10.4274/jcrpe.1194

**Published:** 2014-06-05

**Authors:** Elif Özsu, Gül Yeşiltepe Mutlu, Belkıs İpekçi

**Affiliations:** 1 Kocaeli University Faculty of Medicine, Department of Pediatric Endocrinology and Diabetes, Kocaeli, Turkey; 2 Kocaeli University Medical School, Department of Pediatrics, Kocaeli, Turkey

**Keywords:** Chromosome 13, sexual disorder, microcephaly

## Abstract

Ambiguous genitalia, known to be associated with sex chromosome disorders, may also be seen with autosomal chromosome anomalies. Herein, we report a case with ambiguous genitalia and ring chromosome 13. Ring chromosome 13 is a rare genetic anomaly in which the loss of genetic material determines the clinical spectrum.

## INTRODUCTION

Ambiguous genitalia, a disorder of sex development (DSD) known to be associated with sex chromosome disorders, may also be associated with autosomal chromosome anomalies. Herein, we report an 11-month-old patient with ambiguous genitalia and ring chromosome 13. Ring chromosome 13 is a rare genetic anomaly in which the loss of genetic material determines the clinical spectrum. 

## CASE REPORT

**Figure 1:**
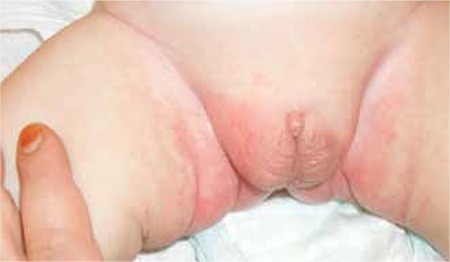
Bifid scrotum and micropenis

**Figure 2:**
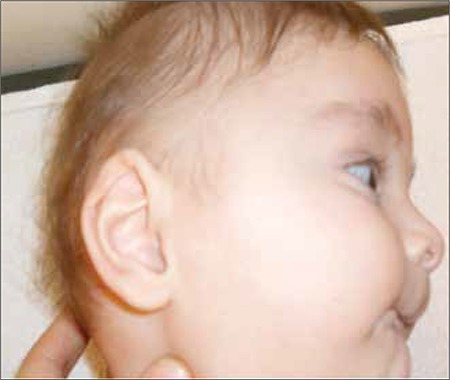
Microcephaly and large ears

This 11-month-old boy was referred to our pediatric endocrinology outpatient clinic because of ambiguous genitalia. He was born after a full-term uneventful pregnancy. His birth weight was 2730 g. He was the second child of non- consanguineous parents and was reared as a male. At presentation, his height was 74.5 cm [0.4 standard deviation score (SDS)], weight was 8.3 kg (-0.6 SDS) and head circumference was 41 cm (-3.2 SDS). Body proportions were normal for his age. He had microcephaly, frontal bossing, a depressed nasal bridge, anteverted nostrils, low-set big auricles and a high arcuated palate (Figure 1). There were two cafe-au-lait spots 2x1 cm in size on his neck and on his lumbar region. Both testicles palpated in the bifid scrotal folds were 2 mL in size. The penis was very small - stretched penile length (SPL) was 10 mm. There was a single urogenital meatus opening in the tip of the penis (Figure 2). Physical examination revealed no other noteworthy findings. 

Neuromotor development was retarded; the patient was diagnosed with autism and development-oriented support was given to him and his family. 

Laboratory tests (hematological investigations, liver function and renal function tests) were normal for his age. Pelvic ultrasound, echocardiogram and cranial magnetic resonance imaging findings were also normal. Serum hormone levels are given in Table 1. Human chorionic gonadotropin (HCG) stimulation test was performed with 1500 U/day for 3 days and stimulated ratio of testosterone/ dihydrotestosterone was found as 11. Serum testosterone level was 435 ng/dL after stimulation with HCG, showing a normal testosterone response. There was also an increase in the SPL (from 12 mm to 24 mm) after HCG stimulation test. 

Karyogram analysis with Tripsin Giemsa Chromosome banding revealed 46 chromosomes including a ring chromosome 13 [46XY r(13)p11q34 (83%)/45XY r(13) (17%)].

## DISCUSSION

Herein, we reported an infant with ring chromosome 13, who presented with microcephaly, dysmorphic features, genital abnormalities and developmental delay. Microcephaly, neuromotor retardation and micro-genitalia are the major clinical findings in patients with ring chromosome 13.

Ring chromosome 13 was first described in 1968 and the characteristic features of these patients are similar to those of 13q deletion carriers (1). It is a rare chromosomal disorder where genetic material from one or both ends of chromosome 13 is missing and the two broken ends have rejoined to form a ring. Clinical findings are correlated with the amount of missing genetic material. However, the phenotype-genotype correlation has not been identified clearly. 

Nieburh and Ottosen described three different ring chromosome 13 syndromes according to the location of the defects in the long arm of the 13th chromosome, during the formation of the ring chromosome. The first group due to loss of the segments 13q34 and 13q33 was characterized by severe mental retardation, hypertelorism, microcephaly, frontal bossing, depressed nasal bridge, significantly protruding incisor teeth and large ears. The second group which resulted from the loss of segments 13q32 and 13q31 was characterized, in addition to the findings in the first group, by aplasia of the thumbs and feet, genital abnormalities, anal atresia and ocular malformations. Retinoblastoma was reported as the characteristic finding in the third group, a finding which resulted from the loss of segment 13q21 (2). The clinical features of our patient (microcephaly, frontal bossing and depressed nasal bridge) are consistent with the first group. 

Patients showing deletions involving q34 were reported to have ambiguous genitalia or hypospadias. However, some cases with interstitial deletion of 13q, where q34 was not deleted, do not show ambiguous genitalia. These findings indicate that 13q34 has genes which are important for genital development (3).

Guala et al (4) described a critical region on chromosome 13, the deletion of which causes a complex phenotype that overlaps with XK syndrome. This syndrome is characterized by multiple congenital abnormalities including facial abnormalities, agenesis of the corpus callosum, aprosencephaly, limb malformations, cardiac defects, genital abnormalities and mental retardation. Chromosome 13 pathology, especially 13 ring chromosomes, was found in five of nine patients with XK syndrome.

Recently, a case of ring chromosome 13 with hypothyroidism has been reported. The patient had primary hypothyroidism and this was the first reported case of such coexistence. Elevated TSH levels may also be associated with brachial arc defects, but this finding could be a coincidence (5). In our patient, TSH level was mildly elevated. However, free thyroxine level was normal and TSH level had decreased to normal levels in the follow-up. Thyroid ultrasound results were normal and there were no findings indicating an association with brachial arc abnormality.

In conclusion, the findings in our patient show that ambiguous genitalia may be a result not only of sex chromosome abnormalities but may also be due to autosomal genetic abnormalities such as trisomy 13,18, triploid syndromes and 4p deletion syndrome (6). Karyotyping is substantial to demonstrate autosomal chromosome anomalies as well as sex chromosome anomalies in DSD cases. 

## Figures and Tables

**Table 1:**
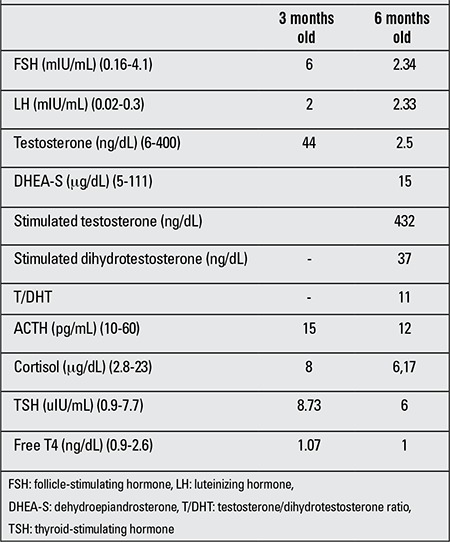
Serum hormone levels in our patient
